# The importance of quality management systems in nuclear medicine departments

**DOI:** 10.1002/jmrs.793

**Published:** 2024-04-20

**Authors:** Kunthi Pathmaraj

**Affiliations:** ^1^ Department of Molecular Imaging and Therapy Austin Health Melbourne Victoria Australia; ^2^ Olivia Newton‐John Cancer Research Institute Melbourne Victoria Australia; ^3^ School of Cancer Medicine La Trobe University Melbourne Victoria Australia; ^4^ RMIT University, School of Health and Biomedical Science Melbourne Victoria Australia

## Abstract

Quality management systems (QMS) in nuclear medicine is an essential component of the Quality program and is instrumental in the safe delivery of a high standard clinical service. The IAEA QUANUM program is a nuclear medicine specific audit program that can be used to assess the standards of a nuclear medicine department and its service delivery. Regular internal and external audits are encouraged as part of the QMS.

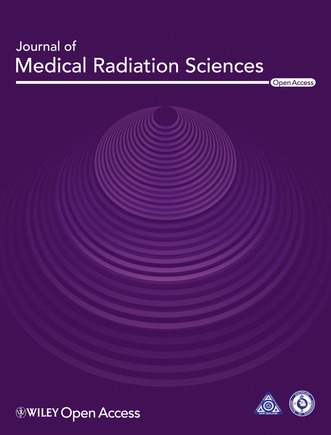

The demand for molecular imaging and radionuclide therapy is growing at an incredible pace worldwide. As nuclear medicine departments get busier, it is imperative that the quality of the diagnostic and therapeutic services is maintained at high standards and that higher throughput does not compromise the standard of patient care. The definition of quality health service by the World Health Organisation (WHO), recognises the need for safe care to protect human rights and takes into account the quality of patient services and patient experience, professional quality and management quality.^1^ High‐quality patient‐oriented health care in molecular imaging and radionuclide therapy requires additional stringent oversight since radiation is an integral part of the service delivery.

The establishment of a quality management system (QMS) should be an essential requirement for a nuclear medicine department (NMD) since it is the QMS that underpins a successful clinical service delivery with inbuilt processes and checks for safety, quality and risk mitigation. The IAEA publication ‘Basics of Quality Management for Nuclear Medicine Practices’ is a useful resource for an NMD wishing to implement or improve a QMS.^2^ An effective QMS will greatly assist in ensuring the safe provision of the clinical services of an NMD, minimising risk to staff, patients and public, as well as providing a mechanism for continuous improvement. The scope of the QMS is not limited to clinical operations but can also support the conduct of clinical trials and research, especially if it is in close conjunction with good clinical practice (GCP).^3^

A QMS should be designed to address the following: (a) an established clinical governance framework to ensure there are systems in place within health service organisations to maintain and improve the reliability, safety and quality of health care (b) a well‐structured documentation system including a quality manual, reliable, tested and established standard operating procedures (SOPs), clearly articulated protocols for the full range of processes in the department and (c) incorporate an established schedule of internal and external audits which serve as quality checks at critical time points.^2^

The paper ‘Quality audits of nuclear medicine practices in a middle‐income African setting’ by Lutaka et al. is an important contribution to the field, and addresses the important and relevant topic of Quality Management and Internal Audits in the practice of nuclear medicine/molecular imaging.^4^ The authors have identified and sought to address a significant gap in nuclear medicine practices in middle‐income country settings in Africa, namely, that there is insufficient data and evidence to demonstrate the existence of robust quality management systems, and regular conduct of self‐assessments that would allow nuclear medicine departments to assess the efficiency of their performance and clinical service provision.

The International Atomic Energy Agency (IAEA) has developed and made available the Quality Management Audits in Nuclear Medicine (QUANUM) program.^5^ QUANUM is an internationally accepted nuclear medicine‐specific quality program that supports the implementation of QMS and the practice of self‐assessment, internal audits and external audits in an NMD. The QUANUM program helps nuclear medicine departments to verify the status of their nuclear medicine practices and benchmark their practice against international reference standards. The QUANUM audit methodology is a two‐step process, the first step being an internal audit by staff members of the institution and the second step involving a voluntary request by the institution to the IAEA for an external audit.^6^ The QUANUM Excel tool is used during both internal and external audits to audit across all processes in an NMD, a radar plot is generated for visual interpretation of results and levels of conformances are calculated. The IAEA published findings of a retrospective analysis covering 42 QUANUM audit missions carried out in 39 centres, from 2008 to 2016, that found the QUANUM program can be applied in a variety of nuclear medicine practices, irrespective of geographical area and socioeconomic conditions.^7^ Interestingly, a similar pattern of conformances and non‐conformances can be observed in both the aforementioned publication and the publication by Lutaka et al., namely, that a high degree of conformances was noted for radionuclide therapy services and scanner QC, and a lower degree of conformances was noted for managerial, documentational aspects, radiation safety and radiation protection aspects of the nuclear medicine services.^7^

The research conducted by Lutaka et al. has closely followed the guidelines stipulated by the QUANUM program and used the QUANUM tools, hence the results of the research can be interpreted confidently against a validated framework. It is encouraging to note that critical aspects of a nuclear medicine service such as the conduct of radionuclide therapy and diagnostic imaging procedures scored relatively highly on the radar plots for most of the departments that took part in this project. There was a reasonable degree of overall conformances noted for the three sites that participated in the project. The self‐audit has also identified many areas of non‐conformances but it must be noted that it is not an uncommon finding during QUANUM audits to identify non‐conformances such as lack of strategic plans for global activities and policies at a departmental level, inadequate SOPs, sub‐optimal levels of radiation monitoring of staff, inadequate monitoring of radiation contamination and lack of opportunities for continuous professional development (CPD).^7^ Hopefully, these findings enable the nuclear medicine departments that participated in this project to secure the required financial, infrastructure, education and training support in order to address the gaps identified by the self‐assessment audits, so that they can elevate their practice to higher and safer standards.

External peer review audits such as the IAEA QUANUM audits can complement a department's internal audits. Results of the internal and external audits should be analysed so that strengths and weaknesses of departmental processes can be recognised. A quality audit process must be patient‐oriented, systematic and evidence based.^8^ It should follow a typical PDCA (plan, do, check, act) process that includes regular planning, review/audit of work practices across all areas in the department such as administrative tasks, patient journey, radiopharmaceutical production and quality control (QC), scanner QC, patient imaging, reporting of the scan and distributions of the results. Auditing every aspect of a patient journey for a procedure that is undertaken by an NMD provides relevant feedback on the operations of the department at various levels.^9^ Patient journey audit tools have been made available by the IAEA to facilitate patient journey audits in an NMD.^10^

In 2015, the Society of Nuclear Medicine and Molecular Imaging (SNMMI) Technologist Section (TS) conducted a large internet survey across 27,989 e‐mail contacts, as part of the multiyear quality initiative by the SNMMI‐TS to help prepare the technologist workforce for an evidence‐based healthcare delivery system that focuses on quality.^11^ It found that most technologists perceive quality to be related to image quality and QC and found a gap in the degree of understanding related to the definition of quality, its measurement, and methods to achieve it. The SNMMI‐TS is planning on rolling out educational programs to address these gaps. Specific parts of the methodology (survey questions) described in this paper can be adapted by other nuclear medicine departments to conduct patient journey audits as part of the QMS.

In nuclear medicine practice, radiation safety poses the greatest risks to not only patients and staff^12^ but also the public. Therefore, the formulation and implementation of a radiation management plan is a critical component of the QMS in an NMD. The radiation management plan should focus on key aspects such as licensing and regulations for radiation workers, occupational radiation safety and personal radiation dose monitoring, radiation protection of patients including diagnostic reference guidelines, personal protective equipment (PPE), radiation shielding equipment, radioactive waste management, reporting of radiation incidents, quality assurance, calibration of radiation equipment and radiation safety training for staff.^13^

Another important aspect of the QMS is the management of human resources, including employing skilled staff, establishing a training and credentialing program for new staff and supporting continuing professional development.

Consideration should be given to how an NMD will implement the requirements of the QMS it has established. Checklists, spreadsheets and mini databases are often used as instruments to record self‐assessment exercises. For example, the QUANUM program provides the QUANUM Excel tool, which includes detailed checklists, to review clinical service provision, radiochemistry and radiopharmacy operations, radiation safety, quality assurance (QA) or QC of instruments and human resource management.^5^ The results recorded in such a checklist can be used to establish the minimal requirements for an NMD to be conformant to internationally recognised quality standards for the covered areas.

In conclusion, the findings of the research conducted by Lutaka et al.^4^ demonstrate the effective use of the QUANUM program and QUANUM tools to gain an understanding of the performance of individual departments and highlights the need for similar research and self‐assessments to be conducted by more nuclear medicine departments. Implementation of a reliable QMS, regular self‐assessments and internal audits are essential for a nuclear medicine department to continuously improve its practice, maintain optimal patient care and maintain a high‐performance workforce. The SNMMI‐TS initiative demonstrates the importance of education around Quality to improve nuclear medicine practices. The IAEA has made several useful resources available on the human health campus website that can be readily accessed and used effectively by nuclear medicine departments. Internal and external audits are instrumental for an NMD because the findings can be used to highlight and justify to its hospital management, the resources the department requires to elevate its practice to international best practice standards.

## Conflict of Interest Statement

The author declare no conflict of interest.

## Data Availability

Data sharing not applicable to this article as no datasets were generated or analysed during the current study.
